# Globotriaosylsphingosine Accumulation and Not Alpha-Galactosidase-A Deficiency Causes Endothelial Dysfunction in Fabry Disease

**DOI:** 10.1371/journal.pone.0036373

**Published:** 2012-04-30

**Authors:** Mehdi Namdar, Catherine Gebhard, Rafael Studiger, Yi Shi, Pavani Mocharla, Christian Schmied, Pedro Brugada, Thomas F. Lüscher, Giovanni G. Camici

**Affiliations:** 1 Heart Rhythm Management Centre UZB, Brussels, Belgium; 2 Clinic for Cardiology, University Hospital of Zurich, Zurich, Switzerland; 3 Cardiovascular Research, Physiology Institute, University of Zurich, Zurich, Switzerland; 4 Center for Integrative Human Physiology, University of Zurich, Zurich, Switzerland; The Chinese University of Hong Kong, Hong Kong

## Abstract

**Background:**

Fabry disease (FD) is caused by a deficiency of the lysosomal enzyme alpha-galactosidase A (GLA) resulting in the accumulation of globotriaosylsphingosine (Gb3) in a variety of tissues. While GLA deficiency was always considered as the fulcrum of the disease, recent attention shifted towards studying the mechanisms through which Gb3 accumulation in vascular cells leads to endothelial dysfunction and eventually multiorgan failure. In addition to the well-described macrovascular disease, FD is also characterized by abnormalities of microvascular function, which have been demonstrated by measurements of myocardial blood flow and coronary flow reserve. To date, the relative importance of Gb3 accumulation versus GLA deficiency in causing endothelial dysfunction is not fully understood; furthermore, its differential effects on cardiac micro- and macrovascular endothelial cells are not known.

**Methods and Results:**

In order to assess the effects of Gb3 accumulation versus GLA deficiency, human macro- and microvascular cardiac endothelial cells (ECs) were incubated with Gb3 or silenced by siRNA to GLA. Gb3 loading caused deregulation of several key endothelial pathways such as eNOS, iNOS, COX-1 and COX-2, while GLA silencing showed no effects. Cardiac microvascular ECs showed a greater susceptibility to Gb3 loading as compared to macrovascular ECs.

**Conclusions:**

Deregulation of key endothelial pathways as observed in FD vasculopathy is likely caused by intracellular Gb3 accumulation rather than deficiency of GLA. Human microvascular ECs, as opposed to macrovascular ECs, seem to be affected earlier and more severely by Gb3 accumulation and this notion may prove fundamental for future progresses in early diagnosis and management of FD patients.

## Introduction

Fabry disease (FD) is an X-linked lysosomal storage disease secondary to deficiency of the lysosomal enzyme alpha-galactosidase A (GLA) where globotriaosylsphingosine (Gb3) – the primary GLA substrate – accumulates in a variety of tissues [1]. While GLA deficiency was always considered as the fulcrum of the disease, in the last decade the main focus of attention shifted towards studying the mechanisms through which Gb3 accumulation – the main offending metabolite – leads to multiorgan failure as observed in FD. Premature multiorgan damage occurs in patients with FD most frequently on the grounds of endothelial dysfunction caused by accumulation of Gb3 in vascular endothelial cells [2]. In addition to the well-described macrovascular disease, FD is also characterized by microvascular function abnormalities, which have been documented by measurements of myocardial blood flow and coronary flow reserve [3], [4]. Furthermore, a survey of female patients with FD revealed that cardiac ischemia could be confirmed by electrocardiographic changes and serological markers in the absence of epicardial coronary artery stenosis, suggesting that ischemia in these patients was of microvascular origin [5]. While a mouse model of GLA deficiency has facilitated the study of glycosphingolipid metabolism abnormalities on macrovascular end points [6] the mechanisms by which GLA deficiency causes microvascular dysfunction remain to be defined.

Hence, this study focuses on analyzing the regulation of key mediators of endothelial function following Gb3 loading or GLA silencing. Additionally, to elucidate differences between macro- and microvascular endothelium, human macro- and microvascular cardiac endothelial cells were used in parallel.

## Materials and Methods

### Cells

Human cardiac microvascular endothelial cells (HMiVECs) and macrovascular endothelial cells (HMaVECs) (Clonetics, Allschwil, Switzerland) were cultured in EGM-2 medium containing 2% or 10% FBS, respectively, and the supplements given by the manufacturer. Cells were grown to confluence in 3 cm dishes and rendered quiescent for 48 hours before stimulation with 5 ng/mL TNF-α. Cells were pretreated with Gb3 (10^−4^ M) for 48 hours. Cytotoxicity was assessed with a colorimetric assay to detect lactate dehydrogenase release (Roche, Basel, Switzerland).

### Gb3-preparation and loading

An aliquot of Gb3 (Matreya, Pleasant Gap, PA) dissolved in chloroform and methanol was air-dried and resuspended in DMSO. The resulting compound was then heated at 90°C for 10 min with occasional vortexing. Finally, an appropriate amount of fatty acid-free bovine serum albumin (BSA, Sigma), dissolved in phosphate buffered saline (PBS) was added to achieve a 1∶1 molar ratio of Gb3/albumin complex. The complex was then sonicated for 5 minutes in a water bath sonicator (Transsonic 460, Elma). Finally, the complex was diluted into EGM medium (Lonza), containing 0.5% FCS. Cultures of endothelial cells were incubated with the medium containing Gb3/albumin complex for 48 hrs.

### Western blot

Protein expression was determined by Western blot analysis as described previously [7], [8]. Antibodies against human ICAM-1 (Invitrogen, Carlsbad, CA, USA) and VCAM-1 (R&D Minneapolis, MN, USA) were used at 1∶1000 and 1∶2000 dilutions, respectively. Antibodies against total eNOS, phosphorylated eNOS, and iNOS (all from Cayman Chemicals, Ann Arbor, MI, USA) were used at 1∶5000, 1∶2000, and 1∶2000 dilutions, respectively. Antibodies against COX-1 and COX-2 (all from Cayman Chemicals, Ann Arbor, MI, USA) were used at 1∶2000, and 1∶1000 dilutions, respectively. Rabbit anti-alpha-galactosidase A polyclonal antibody (Lab Force, Switzerland) was used at 1∶5000 dilution. All blots were normalized to Glyceraldehyde-3-phosphate dehydrogenase (GAPDH; Chemicon, Temecula, CA, 1∶20000 dilution).

### GLA gene silencing through siRNA transfection

Four siRNA sequences specific for human GLA were applied for knocking down GLA expression using the HiPerFect siRNA Transfection System (Qiagen). These siRNA sequences were purchased from Microsynth and were as follows (siRNA 1, 5′-UAAAACCUGCGCAGGCUUCUUTT-3′; siRNA 2, 5′-AUCCGACAGUACUGCAAUCUUTT-3′; siRNA 3, 5′-UUGCCAUCAA-UCAGGACCCUUTT-3′; siRNA 4, 5′-GUGUGGGAACGACCUCUCUUUTT-3′). Briefly, cells were transfected with a 45 nM siRNA

HiPerFect transfection solution and incubated with transfection serum-free medium for the following 4 hrs at 37°C. Subsequently, cells were cultured in the serum-containing growth medium for 48 hrs at 37°C.

### Gb3 ELISA

A 96 well plate was coated with BGR23 IgG2b antibody (Seikagaku, Japan, 1∶500 dilution) for 8 h. Then 5% milk buffer was added for 1 h. This was followed by addition of endothelial cell lysate and incubation with mouse IgM DMAB-1 antibody (kindly provided by J.E. Mansson, Sweden) at 1∶100 dilution for 8 h. Subsequently, wells were washed three times with 1% PBS Tween and then a secondary IgM antibody conjugated to horseradish peroxidase was added. Wells were then washed three times and incubated with substrate solution. Finally, colorimetric readout was measured at 450 nm by spectrophotometry. A standard curve with Gb3 was performed to assure that measurements were taken in the linear range of detection.

### Statistics

Data were analyzed using the Mann–Whitney U-test to compare group means. Results are expressed as mean±standard error of mean (SEM). A P-value<0.05 was considered to represent a significant difference.

## Results

### Gb3 loading and GLA gene silencing

**Figure 1:**
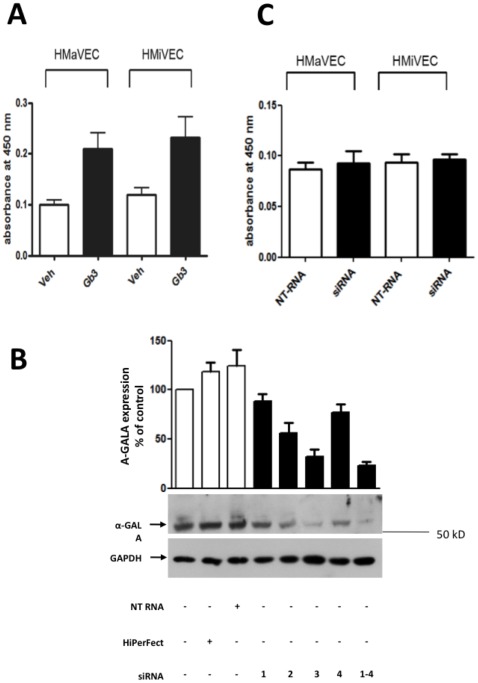
Gb3 loading and alpha-Galactosidase A gene silencing. **A.** Incubation with Gb3 increased intracellular concentration of Gb3, as measured by ELISA, by 2-folds in HMaVECs and HMiVECs (n = 4, p = 0.03 and p = 0.04, respectively, versus untreated control, **B.** Maximal a-Gal A protein silencing of 70% occurred 48 hrs after transfection of HMaVECs with siRNA 3. Transfection of HMaVECs with Non-targeting (NT)-RNA did not alter endothelial a-Gal A expression. Values are representative of at least 4 different experiments; all blots are normalized to GAPDH expression. **C.** Intracellular Gb3 concentrations 2 days following siRNA transfection remained unchanged compared to untreated cells.

Incubation of HMaVECs or HMiVECs with Gb3 increased intracellular concentration of Gb3, as measured by ELISA 2-fold (n = 4, p = 0.03 and p = 0.04, respectively, versus untreated control, **Figure 1A**). To control for potential toxic effects, HMaVECs and HMiVECs were treated with 10^−4^ M Gb3 for 48 hrs. Morphological examination under a light microscope did not reveal any morphological changes (n = 4; shown in data supplement Figure S1).

To downregulate GLA expression in HMaVECs and HMiVECs, siRNA was used. Four different siRNA oligonucleotide sequences on the basis of the human GLA mRNA were designed and applied. A maximal GLA protein silencing of 70% was observed under these conditions (**Figure 1B**) 48 hrs after transfection in the absence of any toxic effect as measured by LDH assay (shown in data supplement Figure S2). Intracellular Gb3 concentrations 2 days following siRNA transfection remained unchanged compared to untreated cells (**Figure 1C**).

### Gb3 impairs eNOS expression in HMiVECs

**Figure 2:**
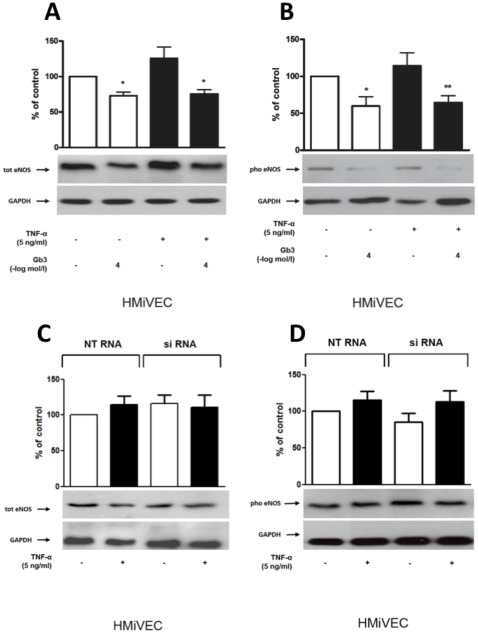
Gb3 impairs eNOS expression in HMiVECs. **A.** Gb3 inhibits total eNOS expression in TNF-α stimulated HMiVECs and under basal conditions. Values are indicated as percent of unstimulated control. *p<0.05 vs unstimulated control; **p<0.05 vs TNF-α alone. **B.** Gb3 inhibits Ser_1177_ pho eNOS expression in TNF-α stimulated HMiVECs and under basal conditions. Values are indicated as percent of unstimulated control. *p<0.05 vs unstimulated control; **p<0.05 vs TNF-α alone. Values are representative of at least 4 different experiments; all blots are normalized to GAPDH expression. **C./D.** In all cases expression remained unchanged following GLA gene silencing.

Gb3 (10^−4^ mol/L) inhibited total eNOS expression in unstimulated as well as TNF-α (5 ng/ml, 5 hrs) stimulated HMiVECs resulting in a 28% and 43% reduction, respectively (n = 7; p = 0.002 and p = 0.001, respectively; **Figure 2A**). In contrast, total eNOS expression in HMaVECs remained unaffected (n = 6; p = NS; shown in data supplement Figure S3). Furthermore, following Gb3 treatment of HMiVECs Ser1177 phosphorylation of eNOS was reduced under both basal conditions (41% reduction, n = 7; p = 0.02; **Figure 2B**) and after stimulation with TNF-α (5 ng/ml); 43% reduction, n = 7; p = 0.03; **Figure 2B**). In contrast, Ser1177 pho eNOS expression in HMaVECs remained unchanged following Gb3 treatment under both baseline conditions and in TNF-α stimulated cells (n = 6; p = NS; shown in data supplement Figure S4). In all cases, expression of both total eNOS and Ser1177 phosphorylated eNOS remained unchanged following GLA gene silencing (**Figure 2C and 2D**).

### Gb3 enhances iNOS expression in HMiVECs

**Figure 3:**
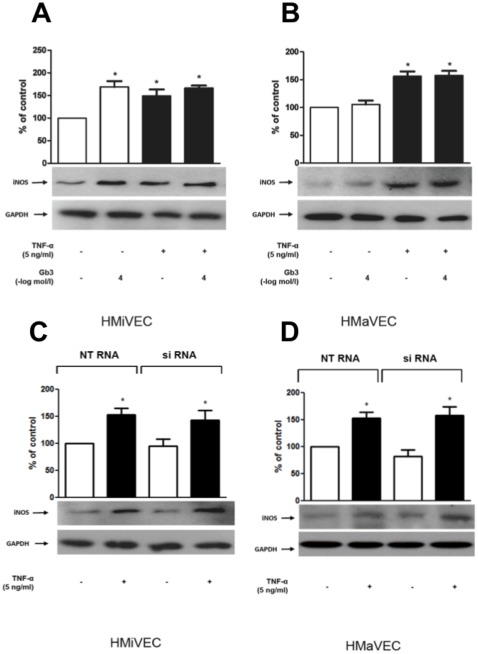
Gb3 enhances iNOS expression in HMiVECs. **A.** Gb3 enhances iNOS expression in HMiVECs under basal conditions. TNF-α enhances iNOS expression in HMiVECs. Values are indicated as percent of unstimulated control. *p<0.05 vs unstimulated control. **B.** TNF-α enhances iNOS expression in HMaVECs. Values are indicated as percent of unstimulated control. *p<0.05 vs unstimulated control; Values are representative of at least 4 different experiments; all blots are normalized to GAPDH expression. **C./D.** GLA gene silencing had no effect on expression of iNOS.

TNF-α (5 ng/ml, 5 hrs) enhanced iNOS expression in HMiVECs by 1.5-fold (**Figure 3A**; p = 0.001). Treatment with Gb3 (10^−4^ mol/L) for 48 hrs enhanced iNOS expression in unstimulated HMiVECs by 1.7-fold, but not in TNF-α stimulated HMiVECs (n = 7; p = 0.0014; **Figure 3A**). TNF-α enhanced iNOS expression in HMaVECs by 1.6-fold (**Figure 3B**; p = 0.009). In HMaVECs iNOS expression was unaffected by Gb3 treatment independent of TNF-α stimulation (n = 7; p = NS; Figure 3B). GLA gene silencing had no effect on expression of iNOS (**Figure 3C and 3D**).

### Gb3 specifically enhances COX-2 but not COX-1 expression in HMiVECs

**Figure 4:**
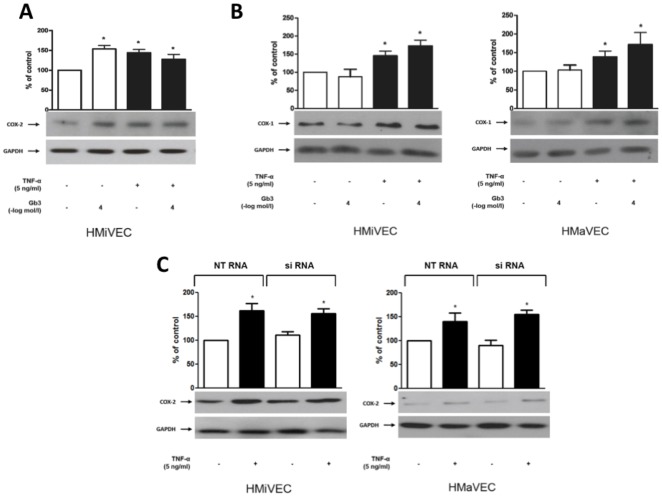
Gb3 enhances COX-2 expression in HMiVECs. **A.** Gb3 enhances COX-2 expression in HMiVECs under basal conditions. TNF-α enhances COX-2 expression in HMiVECs. Values are indicated as percent of unstimulated control. *p<0.05 vs unstimulated control. **B.** TNF-α enhanced COX-1 expression in both HMiVECs and HMaVECs by 1.5-fold and 1.4-fold, respectively. Values are representative of at least 4 different experiments; all blots are normalized to GAPDH expression. **C.** GLA gene silencing had no effect on expression of COX-2.

In unstimulated HMiVECs Gb3 (10^−4^ mol/L) enhanced COX-2 expression by 1.5-fold as compared to controls, but not in TNF-α stimulated cells (n = 7; p<0.0001; **Figure 4A**). Interestingly, in HMaVECs COX-2 expression was not affected by Gb3 irrespective of TNF-α stimulation (n = 7; p = NS, shown in data supplement Figure S5).

TNF-α enhanced COX-1 expression in both HMiVECs and HMaVECs by 1.5-fold and 1.4-fold, respectively (n = 4; p = 0.04 and p = 0.03, respectively; **Figure 4B**). COX-1 expression remained unchanged in HMaVECs and HMiVECs following Gb3 treatment (n = 4; p = NS, **Figure 4B**). Again, GLA gene silencing had no effect on expression (**Figure 4C**).

### Gb3 enhances VCAM-1 but not ICAM-1 expression in TNF-α stimulated HMiVECs

**Figure 5:**
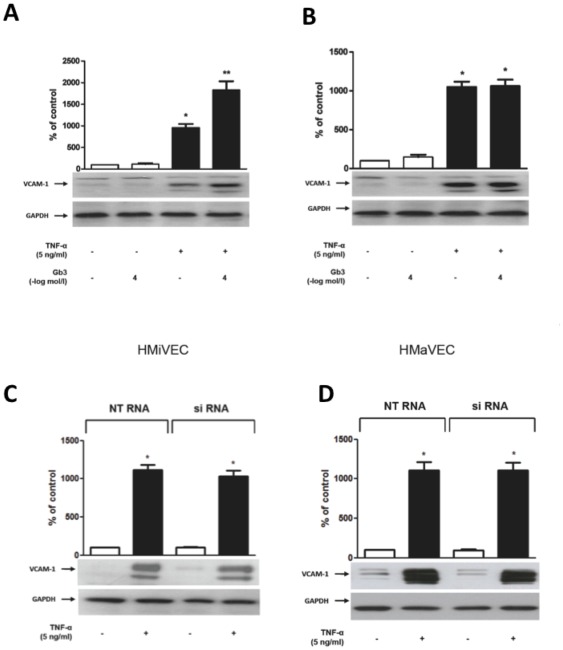
Gb3 enhances VCAM-1 expression in TNF-α stimulated HMiVECs. **A.** TNF-α enhances VCAM-1 expression in HMiVECs. Values are indicated as percent of unstimulated control. *p<0.05 vs unstimulated control; Values are representative of at least 4 different experiments; all blots are normalized to GAPDH expression. **B.** Gb3 enhances VCAM-1 expression in TNF-α stimulated HMaVECs and under basal conditions. Values are indicated as percent of unstimulated control. *p<0.05 vs unstimulated control; **p<0.05 vs TNF-α alone. **C./D.** GLA gene silencing did not affect expression of VCAM-1.

TNF-α (5 ng/ml, 5 hrs) enhanced VCAM-1 expression in both HMiVECs and HMaVECs by 9.5-fold and 10.5-fold, respectively (n = 6; p<0.001; **Figure 5A and 5B**). VCAM-1 expression in HMiVECs was further enhanced by Gb3 by 1.8-fold as compared to TNF-α alone (n = 6; p = 0.0022; **Figure 5A**) while Gb3 did not change TNF-α stimulated VCAM-1 expression in HMaVECs (n = 6; p = NS; **Figure 5B**). ICAM-1 expression in both HMaVECs and HMiVECs remained unchanged following Gb3 treatment with or without TNF-α stimulation (n = 4; p = NS, shown in data supplement Figure S6). As shown in **Figure 5C and 5D**, GLA gene silencing did not affect expression of VCAM-1.

## Discussion

The experimental data gathered in this study contribute two important novel messages to a better understanding of the mechanisms involved in FD's vasculopathy. First, the impairment of endothelial function in FD patients is most likely caused by Gb3 as a consequence of intracellular accumulation, and not through a pleiotropic phenomenon based on deficiency of GLA, as demonstrated by our experiments comparing Gb3 loading to GLA silencing. Hence, our results yield a mechanistic substitute for hitherto existing evidence on FD-associated vasculopathy mainly based on histopathology and imaging studies. Second, our data demonstrate an enhanced sensitivity of HMiVECs to Gb3 accumulation, compared to HMaVECs, as shown by the deregulation of key vasoactive pathways such as eNOS and COX occurring primarily in HMiVECs. These findings support previously observed clinical findings hinting an early and, often initially silent involvement of the microcirculation in FD patients.

We selected a novel experimental setting so as to assess comprehensively the effect of Gb3 accumulation with and without a downregulation of GLA expression in human endothelial cells. To this end, we purposely chose a time point after GLA silencing, which was early enough so that it would not result in increased Gb3 levels. Indeed, an eventual Gb3 accumulation following GLA silencing would have rendered our experimental setup inappropriate. The functional role of GLA in cultured cells has been addressed in an earlier report where the authors showed twofold increase in Gb3 expression when GLA was knocked down for 2 months and, most importantly, stated that in shorter times (such as those in our experiments) it is reasonable not to see a spontaneous accumulation of Gb3 following GLA silencing [9]. Thus, we were able to establish a reproducible silencing of GLA using siRNA and also exclude any toxic effect of the GLA silencing which would have confounded our results substantially. The same holds true for the evidence of Gb3 accumulation and toxicity in Gb3 treated cells. It should be emphasized – although it has been repeatedly indicated that the storage is exclusively in lysosomes and the disease process rather mechanical [10] – that several studies demonstrated Gb3 being not only present in lysosomes, but rather widely distributed in other cellular structures including the endoplasmatic reticulum, cell membrane, and nucleus [11]. Accordingly, there is increasing evidence suggesting that the wide intracellular accumulation of Gb3 brings forth dysfunction of other cellular key components involved in the regulation of endothelial function [12], [13]. Our observation was in fact, that total eNOS and pho-eNOS, both associated with the cell membrane, are downregulated in Gb3 loaded HMiVECs cells, while iNOS expression was upregulated. The observed dysregulation of the L-arginine/nitric oxide (NO) pathways in HMiVECs cells may adversely affect vascular function and blood flow to vital organs, thus contributing to the risk of an incipient vasculopathy and provide a pathologic milieu for the accelerated development of cardiovascular complications. Interestingly, such deregulations of endothelial mediators were not present in GLA silenced cells, regardless of whether HMiVECs or HMaVECs were used. Indeed, expression of eNOS and iNOS was unchanged under these conditions, thus underlining a key role of Gb3. While our study may be the first detailed analysis comparing the effects of GLA silencing versus Gb3 loading in endothelial cells, previous studies focusing on eNOS and iNOS activity in the setting of a FD associated vasculopathy support our current findings [12], [13], [14], [15], [16].

Another group of enzymes, which are crucially involved in the regulation of endothelial function are the cyclooxygenase isoforms COX-1 and COX-2. We have demonstrated that COX-2 expression is enhanced in Gb3 treated HMiVECs, while COX-1 expression remains unaffected, suggesting that upregulation of vasoconstrictive COX-2 derivates may contribute to microvascular dysfunction also in FD, as previously described for other experimental settings [17]. Generally, it has been demonstrated that endothelium-dependent contractions particularly in arterial hypertension and diabetes mellitus are COX-dependent and that both COX-1 and COX-2 activity can mediate or enhance endothelium-dependent contractions [18]. Of note, COX-2 can act as a major source of the vasodilator PGI_2_, particularly under conditions when NO bioavailability is diminishing [18]. Furthermore, it has been reported that receptor-induced endothelium-dependent contractions were impaired in aortas of GLA knockout mice, but could be restored by the nonspecific COX-inhibitor indomethacin [19]. The current evidence on the aberrant vascular reactivity in FD in this context can be attributed in part to changes in endothelial COX-1 and COX-2 activity. However, COX-1 and COX-2 appear to contribute to the vasculopathy in different ways, e.g., specific inhibition of COX-1 improved endothelium-dependent relaxation in aortas from GLA knockout mice, whereas COX-2 inhibition exacerbated the endothelial dysfunction [19]. Indeed, COX-2-derived prostaglandins can play a compensatory role for the decreased NO bioavailability [20], [21], thus possibly explaining some of the detrimental cardiovascular effects associated with COX-2 inhibitors [22]. Additionally, COXs are also involved in the endothelial generation of reactive oxygen species, a key factor in the generation of endothelium-dependent contractions [23], [24]. Whether these effects can be translated into the vasculopathy in FD is still a matter of debate and not entirely clear. Silencing of GLA did not lead to any significant changes in COX-1 or COX-2 expression in either HMiVECs or HMaVECs underscoring once again a pivotal role of Gb3 levels in affecting expression of key vasoactive enzymes.

The expression of adhesion molecules is an early step in vascular disease both experimentally and in humans [25], [26]. Indeed, the plasma levels of the soluble form of such proteins are positively related to outcome [26]. In this context, it is of interest that we found that VCAM-1, but not ICAM expression is upregulated following Gb3 treatment only in HMiVECs, suggesting that Gb3 induces a pro-inflammatory phenotype in FD. Increased plasma levels of other endothelial atherosclerotic factors and leukocyte adhesion-molecule expression have recently been demonstrated in FD supporting our findings and directing towards a better understanding of clinical manifestations [27], [28], [29]. Correspondingly, cardio- and cerebrovascular complications like cerebral white matter lesions are frequently found in FD, mainly attributed to thrombo-embolic or ischemic events [30], [31], [32].

In summary, our findings add to the current evidence on the mechanisms of vascular complications and their clinical sequelae in patients with FD. Indeed, FD patients have a substantially limited life expectancy which takes its onset in early and extensive damage to cardiovascular tissues. However, the pathophysiology of this specific vasculopathy is far from being understood. Our findings demonstrate that the phenotype of microvascular endothelial cells is specifically altered by the intracellular accumulation of Gb3 leading to a vasoconstrictive and pro-inflammatory phenotype. Of note, we demonstrate that such changes do occur due to a direct effect of Gb3, and not of GLA. The fact that the effects of Gb3 accumulation was primarily seen in micro- and not in macrovascular endothelium is consistent with previous observations that atherosclerosis as such is not a consistent finding in FD [33]. Our data underscore an enhanced susceptibility of HMiVECs to Gb3 accumulation compared to HMaVECs. Although longer exposure to Gb3 may result in similar effects also in HMaVECs, the fact that HMiVECs manifest an earlier deregulation most probably resulting in microvascular dysfunction is an important hint with respect to the early pathophysiological changes, which may take place in FD patients. It is thus conceivable that cardiac microvascular dysfunction maybe a landmark exam for early diagnosis and treatment of FD patients. Other reports suggest that smooth muscle proliferation in the arterial media layer may also contribute to the vasculopathy seen in FD [34], [35].

For the time being, treatment with enzyme replacement therapy (ERT) is the best option for patients in developed countries. Recombinant GLA is presumed to be removed from the systemic circulation and subsequently transported to its site of action in cellular lysosomes and, most importantly, not in the cytosol, as no reversibility of tissue uptake or release into the circulation occurs and the enzyme is only activated by the low pH before its subsequent degradation in the lysosomes [36]. It has been shown that ERT – given intravenously – results in a reduction of endothelial Gb3 content [37]. However, not all patients, in particular those with an advanced stage of the disease, benefit from ERT. The latter might at least in part be explained by the fact that ERT – although unfolding its full potential in the lysosomes – stops further extralysosomal and thus pathogenic Gb3 accumulation in line with still intact autoprotective cellular processes before some kind of “point of no return” of cellular damage occurs.

We show indeed, that the enzyme deficiency as such is not a major contributor to the pathophysiology, which is strongly backed up by the fact that female carriers may suffer from vascular complications as well. In fact, females and patients with an advanced stage of the disease do not show changes in endothelial Gb3 content as a surrogate marker to the clinical course under ERT [38], raising the question whether ERT should be started as early as possible in order to halt the pathophysiologic cascade of the vasculopathy, even before specific clinical manifestations occur and furthermore whether established and thus supplemental treatment options such as anti-platelet therapy and/or lipid lowering/antihypertensive drugs should be indicated for patients with FD should be conceived. We conclude herewith that vasculopathy in FD is directly caused by intracellular Gb3 accumulation primarily in microvascular endothelial cells, while deficiency of GLA alone does not cause any deregulation of key vasoactive mediators. HMiVECs, as opposed to HMaVECs, seem to be affected earlier and more severely by Gb3 accumulation and this notion may prove fundamental for future progresses in early diagnosis and treatment of FD patients.

## Supporting Information

Figure S1 Morphological examination under a light microscope did not reveal any morphological changes (Figure S1).
(TIF)

Figure S2 A maximal GLA protein silencing was observed 48 hrs after transfection in the absence of any toxic effect as measured by LDH assay.
(TIF)

Figure S3 Total eNOS expression in HMaVECs remained unaffected by Gb3 loading.
(TIF)

Figure S4 Ser1177 pho eNOS expression in HMaVECs remained unchanged following Gb3 treatment under both baseline conditions and in TNF-α stimulated cells.
(TIF)

Figure S5 In HMaVECs COX-2 expression was not affected by Gb3 irrespective of TNF-α stimulation.
(TIF)

Figure S6 ICAM-1 expression in both HMaVECs and HMiVECs remained unchanged following Gb3 treatment with or without TNF-α stimulation.
(TIF)
