# Correction: Globotriaosylceramide Accumulation and Not Alpha-Galactosidase-A Deficiency Causes Endothelial Dysfunction in Fabry Disease

**DOI:** 10.1371/annotation/7b2c04df-8592-4fb7-8608-3039db28b504

**Published:** 2012-09-12

**Authors:** Mehdi Namdar, Catherine Gebhard, Rafael Studiger, Yi Shi, Pavani Mocharla, Christian Schmied, Pedro Brugada, Thomas F. Lüscher, Giovanni G. Camici

In our experiments, we used GlobotriaosylCERAMIDE (Gb3) and not - as wrongly stated - GlobotriaosylSPHINGOSINE (lyso-Gb3). Nevertheless, the abbreviation we used, "Gb3", is the correct one and accurate throughout the manuscript.

The instances in the text where the substance's name is mentioned in full should be corrected; more precisely the title, the abstract (background section) and the Introduction section (3rd line). In those three cases, Globotriaosylsphingosine should be replaced by Globotriaosylceramide.

The correct citation is: Namdar M, Gebhard C, Studiger R, Shi Y, Mocharla P, et al. (2012) Globotriaosylceramide Accumulation and Not Alpha-Galactosidase-A Deficiency Causes Endothelial Dysfunction in Fabry Disease. PLoS ONE 7(4): e36373. doi:10.1371/journal.pone.0036373

