# Supplementary figures and images for: Globotriaosylsphingosine Accumulation and Not Alpha-Galactosidase-A Deficiency Causes Endothelial Dysfunction in Fabry Disease
Source: PLoS One. 2012 Apr 30;7(4):e36373. doi: 10.1371/journal.pone.0036373 (PMC3340376; doi:10.1371/journal.pone.0036373)

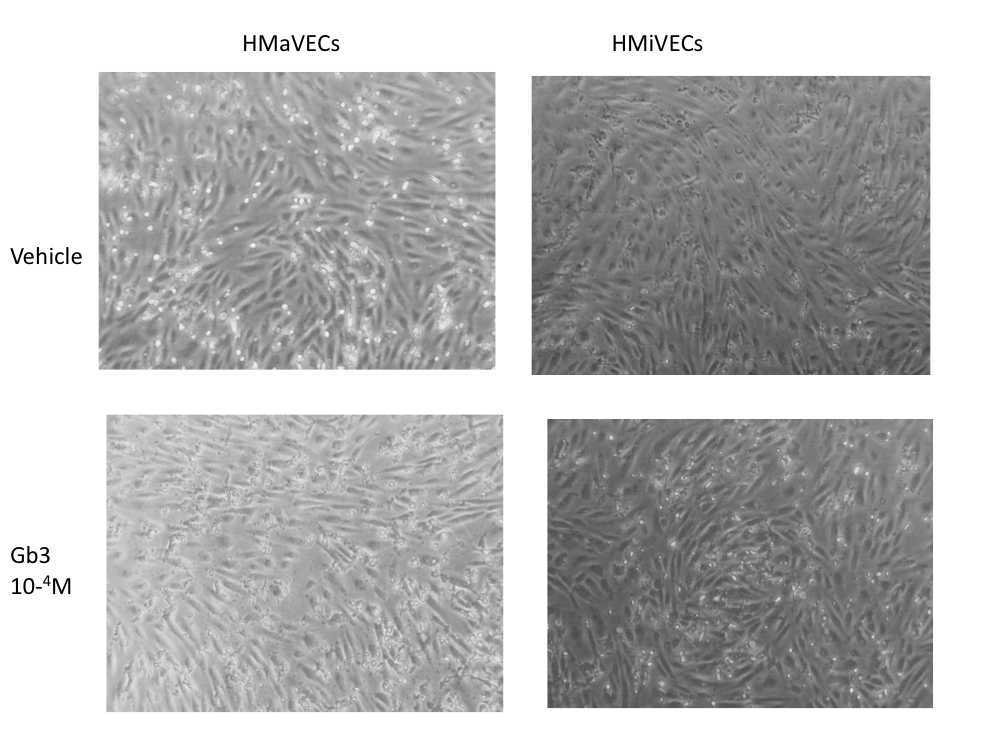

Supplement: Figure S1 — Morphological examination under a light microscope did not reveal any morphological changes (Figure S1). (TIF) [file pone.0036373.s001.tif]

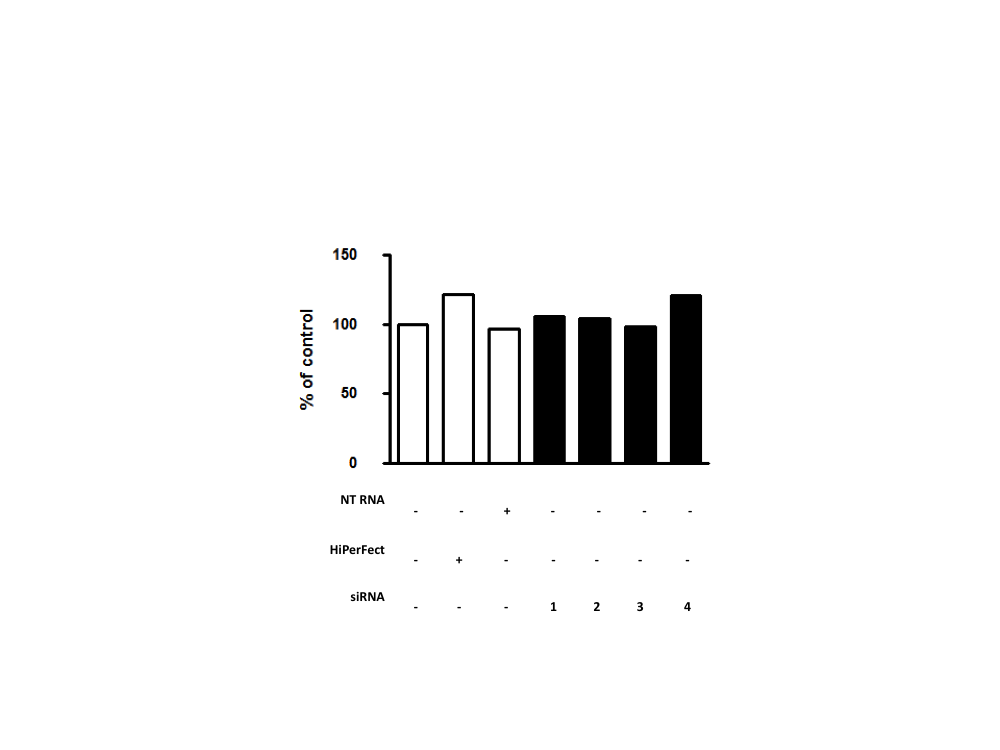

Supplement: Figure S2 — A maximal GLA protein silencing was observed 48 hrs after transfection in the absence of any toxic effect as measured by LDH assay. (TIF) [file pone.0036373.s002.tif]

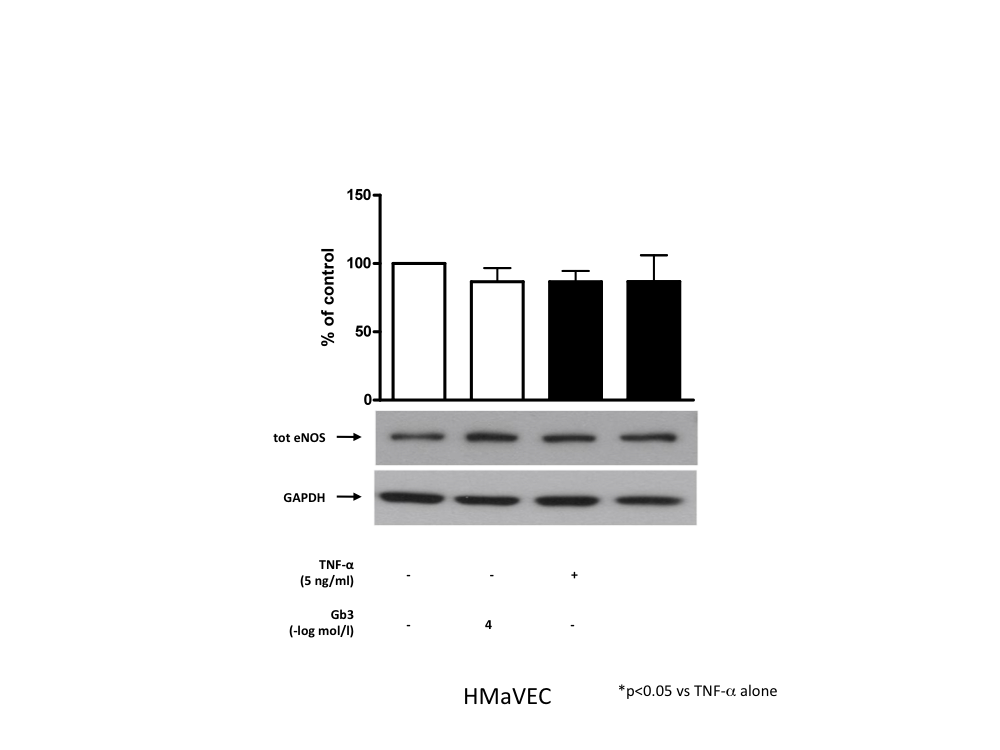

Supplement: Figure S3 — Total eNOS expression in HMaVECs remained unaffected by Gb3 loading. (TIF) [file pone.0036373.s003.tif]

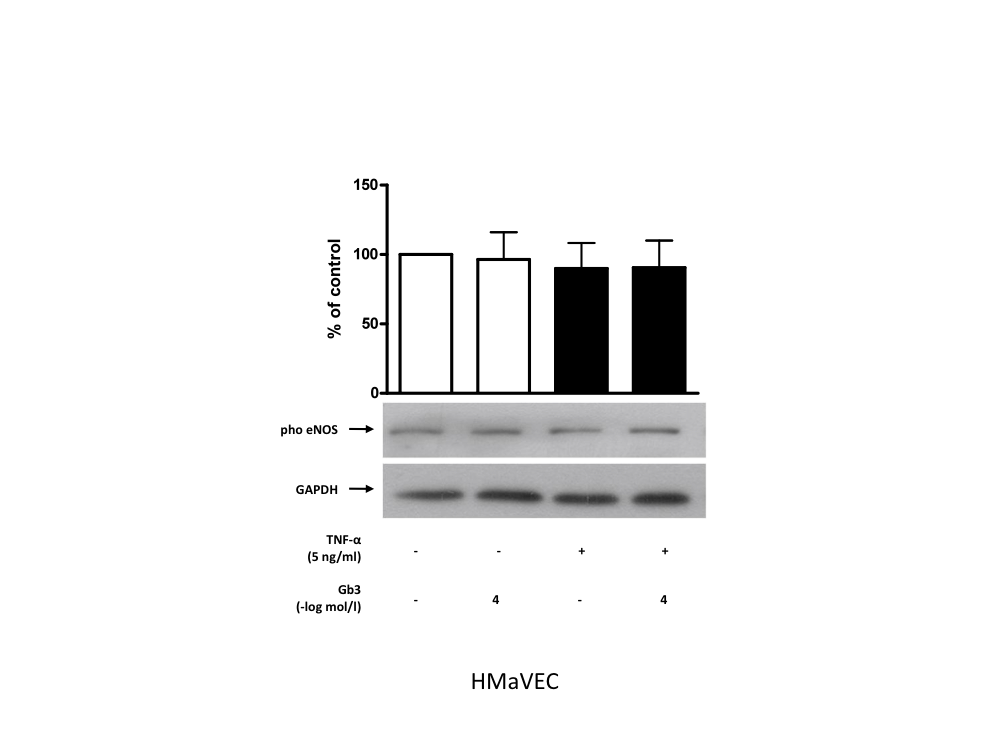

Supplement: Figure S4 — Ser1177 pho eNOS expression in HMaVECs remained unchanged following Gb3 treatment under both baseline conditions and in TNF-α stimulated cells. (TIF) [file pone.0036373.s004.tif]

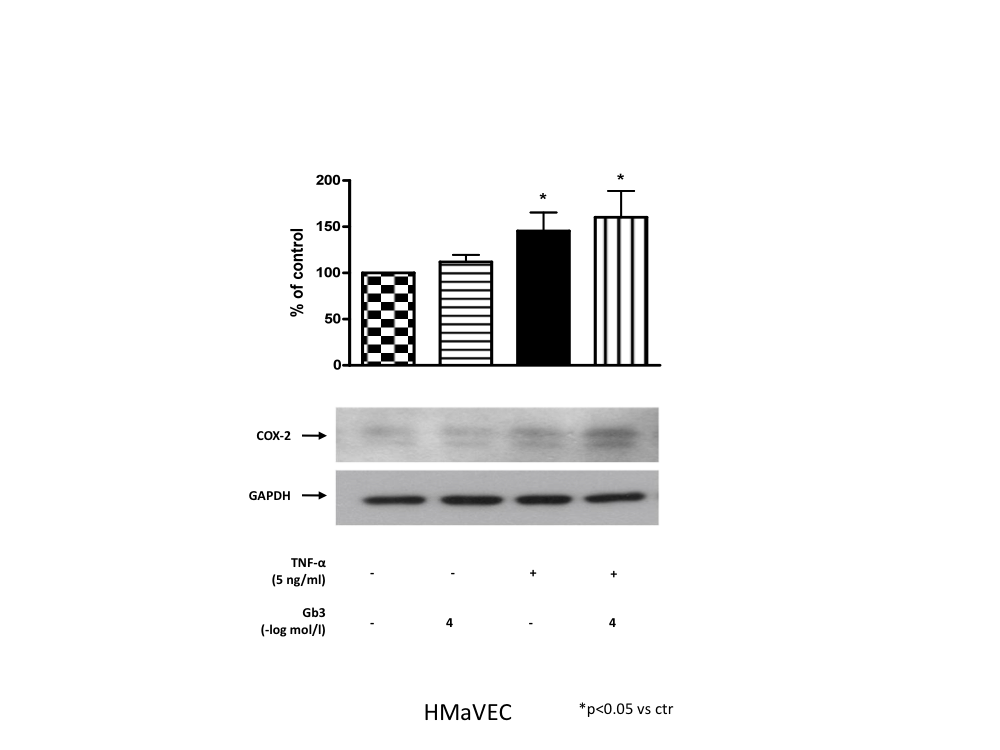

Supplement: Figure S5 — In HMaVECs COX-2 expression was not affected by Gb3 irrespective of TNF-α stimulation. (TIF) [file pone.0036373.s005.tif]

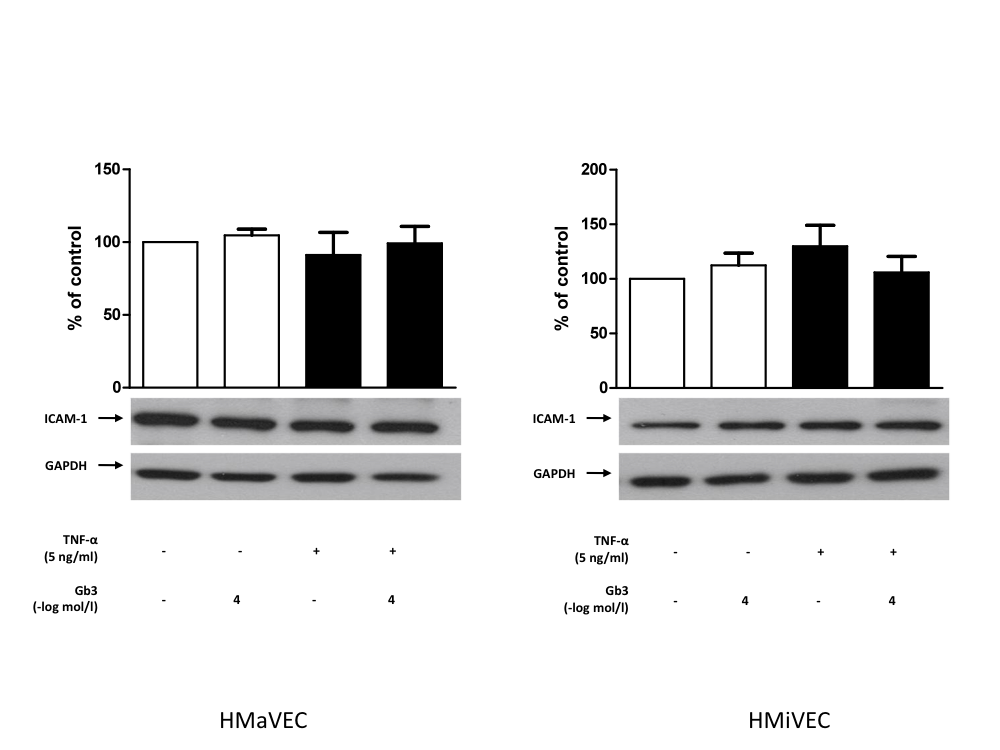

Supplement: Figure S6 — ICAM-1 expression in both HMaVECs and HMiVECs remained unchanged following Gb3 treatment with or without TNF-α stimulation. (TIF) [file pone.0036373.s006.tif]
